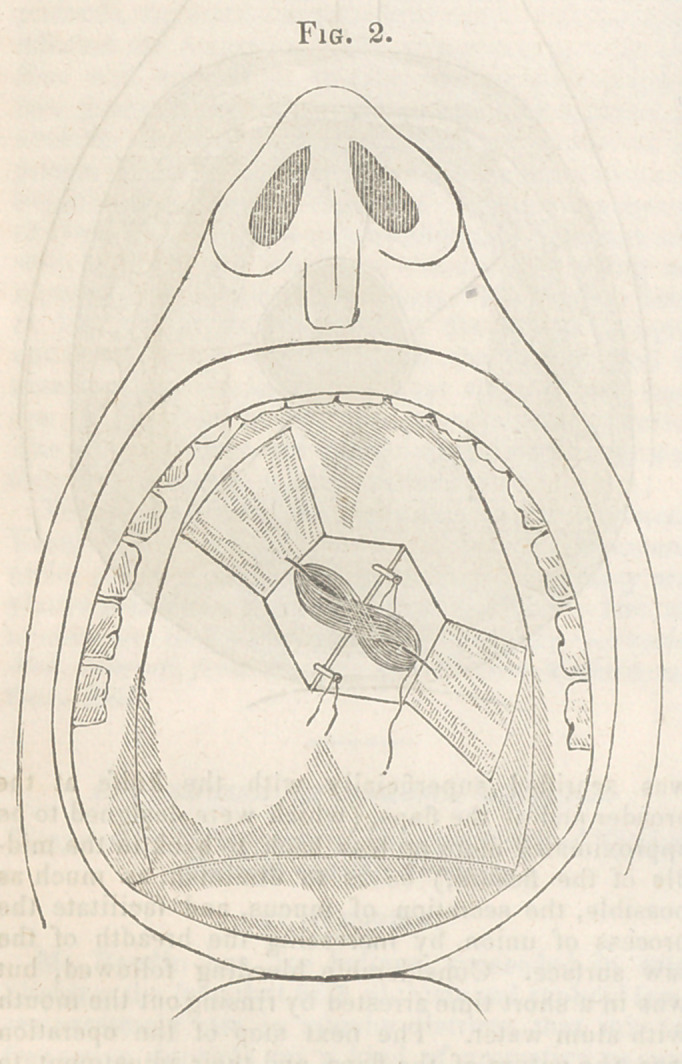# On the Possibility of Closing Fistulous Orifices of the Bony Palate by a Plastic Operation

**Published:** 1844-01-27

**Authors:** J. Pancoast

**Affiliations:** Professor of Anatomy in Jefferson Medical College, Lecturer on Clinical Surgery at the Philadelphia Hospital, &c.


### THE MEDICAL EXAMINER,

### And Record of Medical Science.

Vol. VII.] PHILADELPHIA, SATURDAY, JANUARY 27, 1844. [No. 2.

### ON THE POSSIBILITY OF CLOSING FISTULOUS ORIFICES OF THE BONY PALATE BY A PLASTIC OPERATION.

BY J. PANCOAST, M. D.

Professor of Anatomy in Jefferson Medical College, Lecturer on Clinical Surgery at the Philadelphia Hospital, &c.

Openings through the bony palate, establishing a communication between the mouth and nose, may accrue as the consequence of wounds, caries, necrosis, or syphilitic ulceration. Metallic obturators, or plugs made of prepared sponge, or a roll of linen, or some similar material, have, from the earliest times, been used to close these fistulous orifices, so as to prevent the food from passing into the nostrils, and enable the patient to speak intelligibly. Obvious inconveniences result from all such expedients, which, at the best, but imperfectly fulfil their objects, and occasion an offensive discharge, like that of ozoena. Where the orifice is not too large, but such as to leave room for the formation of flaps from the palatine mucous membrane, a well devised plastic operation for the purpose of making a permanent closure is greatly preferable to either. The process however, is difficult of execution, and the spasmodic cough, which is apt to be produced from the irritation of this sensitive membrane, is, when it occurs, an obstacle in the way of success. The following notes of a case, for which I am indebted to my friend Dr. Wm. McPheeters, of North Carolina, one of the Resident Surgeons of the Philadelphia Hospital, will serve to illustrate the plan of proceeding.

Malek Moore, æt. 30, entered the hospital October, 1840. Eighteen years ago he had an attack of syphilis. Twelve years afterwards he had a second attack followed with secondary symptoms, which destroyed the palatine processes of the upper maxillary bones, near their place of junction with the horizontal portions of the palate bones, and a considerable part of the septum narium, leaving an opening of the size of a ten cent piece between the mouth and nose, materially impairing his speech, and interfering with mastication.

Nov. 28, 1840. Dr. Pancoast performed before the hospital class the following uraniscoplastic or palatoplastic operation, for the closure of this large opening. The patient was seated upright in a chair, facing the light. The surgeon seated opposite to him with a double edged scalpel, strongly curved near the point, marked out with the point of the instrument, and then dissected up two flaps of mucous membrane, of a somewhat triangular shape, one from the anterior and right side of the orifice, the other from the posterior and left side. The base of each flap, which was about three-quarters of an inch in extent, touched the roots of the alveolar processes. The pedicles, or adherent portions of the flap, were about three-eighths of an inch wide, and near the margins of the orifice. (See fig. 1.) The cicatrised margin of the opening was shaved away with the knife. The mucous membrane

Fig. 1.

was scarified superficially with the knife at the broader end of the flaps, (which were designed to be approximated more or less back to back in the middle of the fissure,) so as to diminish, as much as possible, the secretion of mucus, and facilitate the process of union, by increasing the breadth of the raw surface. Considerable bleeding followed, but was in a short time arrested by rinsing out the mouth with alum water. The next step of the operation was the suture of the flaps, and their adjustment to the margins of the orifice. The flaps, when reverted, and their mucous surfaces turned upwards to the nostrils, readily met in the middle line, but it was necessary to confine them against the arched roof of the palate, which was some lines above the plane formed by their junction after they were inverted. To accomplish this object, two long, well waxed, silken ligatures were each armed with a needle at both ends. With a pair of Physick's forceps the needles were passed through the broad end of the flaps, so that the loose ends of each ligature were brought out of the mouth over the raw surfaces of the flaps. The intermediate loops were passed into the eye of a curved probe, and carried from the mouth through the fistulous orifice, and out at the anterior nares. Beneath these loops was next passed the end of a hollow bougie, which was carried into the nostril, so as to lay across the opening communicating with the mouth. The ends of the threads were now drawn on the side of the mouth, and the loops astride of the piece of bougie pushed back till they were over the orifice. The ligatures were then tied in the mouth, forcing up the flaps to the roof of the palate, and bringing them nearly to the level of the bougie. The flap, in order to admit of the subsequent shrinking and contraction, which always follows in plastic operations, and especially when union does not take place by first intention, a result which was hardly to be expected here, were made larger than absolutely necessary to close the opening. They formed, therefore, a keel shaped projection downwards. To make the adjustment of the flaps to the arch of the palate still more perfect, a stiff, well sharpened, semi-circular pin, made of palladium wire, was passed from before backwards through both flaps, with the curve concentric to, and in contact with the arch of the palate. Over this a common twisted suture was made, (as seen in fig. 2,) and the adjustment of

Fig. 2.

the flaps to the raw edge at the margin of the orifice was now rendered perfect. The extremities of the pin were cut off short with the pliers, so as not to irritate the tongue, and the loose ends of the ligature removed. The bougie cut off just in front of the nostril was secured, so as to prevent its sliding. The operation was necessarily somewhat protracted, but was attended with but little suffering.

The patient was put in bed, and kept on his back, fed on gruel, and took occasional doses of Tr. Opii Acetata. For several days every thing promised an immediately successful issue. The bougie became loose on the third day, and was removed; the ligatures remaining. On the fifth day the pin was withdrawn; the flaps seemed to have united everywhere except at the back part, where a small oblique opening was left. On the sixth day a violent spasmodic cough set in, not traceable to any other exposure than rising at night, when not well watched, in a room but illy warmed. The cough was accompanied with symptoms of bronchitis, for which he was cupped, blistered, &c. In one of these paroxysms the union partially gave way, and the ligatures, which were cutting out, were removed. On the subsidence of the bronchial affection the orifice was found diminished one-half in size. On stimulating the edges with a solution of lunar caustic, the opening was still further diminished by granulation, till it was about two-thirds the size of a common writing quill. Beyond this point it would not improve.

A repetition of the former operation on a small scale, the pressure of a well adjusted obturator, acting only around the margin, would in all probability have sufficed to close the orifice completely. But the patient, satisfied with his improved condition, and desirous of securing some occupation, left the hospital; still, however, wearing in the orifice a small pledget of lint during his meals.

On a reexamination of the patient a year subsequently, but little change had seemed to have taken place since the period of his dismissal. The surfaces from which the flaps had been detached, and which were allowed to fill up by granulation, were smooth, and, but for the white aspect of the cicatrised covering, presented a perfectly natural appearance.

## Figures and Tables

**Fig. 1. f1:**
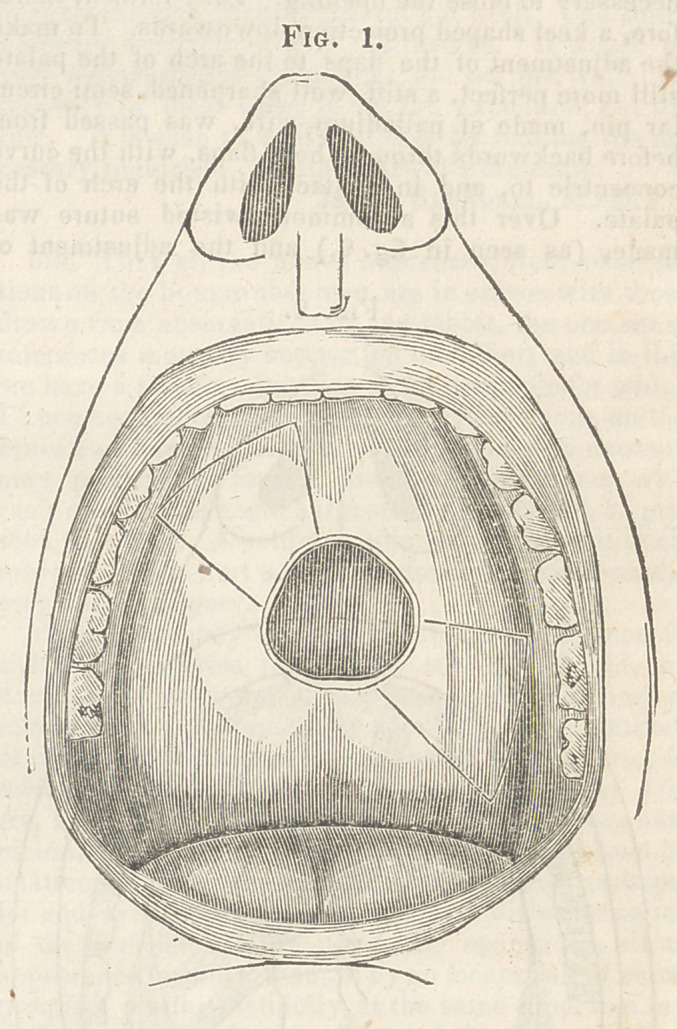


**Fig. 2. f2:**